# Brief temperature stress during reproductive stages alters meiotic recombination and somatic mutation rates in the progeny of *Arabidopsis*

**DOI:** 10.1186/s12870-017-1051-1

**Published:** 2017-06-14

**Authors:** Ramswaroop Saini, Amit Kumar Singh, Shanmuhapreya Dhanapal, Thoufeequl Hakeem Saeed, Geoffrey J. Hyde, Ramamurthy Baskar

**Affiliations:** 1 Department of Biotechnology, Indian Institute of Technology–Madras, Bhupat and Jyoti Mehta School of Biosciences, Chennai, 600 036 India; 2 Write about Research, 14 Randwick Street, Randwick, Sydney, 2031 Australia

**Keywords:** *Arabidopsis*, Temperature stress, Meiotic recombination, Somatic mutation rates, Stochasticity

## Abstract

**Background:**

Plants exposed to environmental stresses draw upon many genetic and epigenetic strategies, with the former sometimes modulated by the latter. This can help the plant, and its immediate progeny, at least, to better endure the stress. Some evidence has led to proposals that (epi) genetic changes can be both selective and sustainably heritable, while other evidence suggests that changes are effectively stochastic, and important only because they induce genetic variation. One type of stress with an arguably high level of stochasticity in its effects is temperature stress. Studies of how heat and cold affect the rates of meiotic recombination (MR) and somatic mutations (SMs, which are potentially heritable in plants) report increases, decreases, or no effect. Collectively, they do not point to any consistent patterns. Some of this variability, however, might arise from the stress being applied for such an extended time, typically days or weeks. Here, we adopted a targeted approach by (1) limiting exposure to one hour; and (2) timing it to coincide with (a) gamete, and early gametophyte, development, a period of high stress sensitivity; and (b) a late stage of vegetative development.

**Results:**

For plants (*Arabidopsis thaliana*) otherwise grown at 22 °C, we measured the effects of a 1 h exposure to cold (12 °C) or heat (32 °C) on the rates of MR, and four types of SMs (frameshift mutations; intrachromosomal recombination; base substitutions; transpositions) in the F1 progeny. One parent (wild type) was stressed, the other (unstressed) carried a genetic event detector. When rates were compared to those in progeny of control (both parents unstressed) two patterns emerged. In the progeny of younger plants (stressed at 36 days; pollinated at 40 days) heat and cold either had no effect (on MR) or (for SMs) had effects that were rare and stochastic. In the progeny of older plants (stressed at 41 days; pollinated at 45 days), while effects were also infrequent, those that were seen followed a consistent pattern: rates of all five genetic events were lowest at 12 °C and highest at 32 °C, i.e. they varied in a “dose-response” manner. This pattern was strongest (or, in the case of MR, only apparent) in progeny whose stressed parent was female.

**Conclusion:**

While the infrequency of effects suggests the need for cautious inference, the consistency of responses in the progeny of older plants, indicate that in some circumstances the level of stochasticity in inherited genetic responses to heat or cold stress can be context-dependent, possibly reflecting life-cycle stages in the parental generation that are variably stress sensitive.

**Electronic supplementary material:**

The online version of this article (doi:10.1186/s12870-017-1051-1) contains supplementary material, which is available to authorized users.

## Background

When exposed to an abiotic or biotic stress, plants can draw upon an extensive repertoire of strategies, genetic and epigenetic, that potentially enable them to better endure that stress, either during their own lifetimes or those of their progeny [1–4]. Understanding how environmental stresses induce (epi) genetic variability not only informs our understanding of how plants evolved in the past [5] and might respond in the future [6], but it could also help in the development of more ‘future-proof’ plant varieties. Within developmental time-frames, responses to stress mobilise epigenetic changes, the most common of which involve the unsilencing of genes via the demethylation of DNA [3]. These epigenetic revisions are often redeployed if the plant experiences the stress again at some later point (i.e. priming, [3, 4]) and indeed may be inherited across several generations, although conclusive evidence of permanent change to the lineage has not yet emerged [7, 8]. Within evolutionary time-frames, stress has the potential to induce genuine, stably inherited genetic change (which could be modulated epigenetically [9]). Many forms of stress have been shown to increase the rates of meiotic recombination (MR) and various somatic mutations (SMs) [10–13], the latter being of relevance because plant gametes do not develop in isolation from the soma. It has even been speculated that plants have evolved the ability to selectively induce mutations in genes associated with a beneficial response to the initiating stress [1, 2]. In contrast, after studying the transgenerational effects of a wide range of stresses on rates of intrachromosomal recombination (ICR), it was concluded that responses in the progeny of stressed plants were “rare and stochastic”, and that it is the genetic variation arising from such stochasticity which is the main, or sole source, of any evolutionary benefits [7].

One of the most important varieties of natural stress that can promote genetic variability in plants is an extreme of temperature. Nevertheless, the effects of heat and cold on the rates of MR and SMs have only been partially described and the findings have not been consistent. MR has been the most studied type of change, either directly or via rates of chiasmata formation, in a range of species. Heat and cold have variously been reported to increase rates [14–17], decrease rates [15, 18], or have no effect [19]. For SMs, where rates been studied in the progeny of stressed parental plants, there is a similar story of variability. Microsatellite instability, particularly, and base substitution rates also, have been reported to respond in a mixed manner to heat and cold [12]. For ICR, one study reported increased rates in response to both heat and cold in *Arabidopsis* [12], while another, using two lines of *Arabidopsis*, found that cold had no effect, and that heat affected only one line, and caused rates to decrease [7]. The responses to heat in the latter report were part of the “rare and stochastic” pattern reported for responses to a variety of stressing agents [7]. While some of the overall variability of these previous studies might be due to studying different species, or some degree of inbuilt stochasticity, the findings of our recent study, wherein the basal rates of some SMs were found to increase with age in some (but not all) lines of *Arabidopsis* [20], indicate the possibility that variability could also emerge from the use of different detector lines, and, more importantly, from exposing the plants to stress at different, potentially non-optimal, stages of development. Some genetic repair mechanisms, at least, become less efficient with age [20–23] and during gametophyte development, the plant is highly sensitive to the effects of stress [24, 25]. If one wishes to understand, and know how to maximise, the net effect of temperature stress on plant genetic variability, these findings, collectively, suggest the benefits of: (1) studying a more inclusive suite of genetic responses, and (2) exposing plants to the temperature stress in a manner that might induce, in their progeny, responses that are more consistent.

In this study using the model plant *Arabidopsis*, we have examined the effects of heat and cold on the rates of five types of genetic change: MR, ICR, transpositions, point mutations (base substitutions) and frame shift mutations (FSM; a measure of microsatellite instability; [26]). We have adopted some refinements to typical temperature treatment protocols that we believed might lead to more consistent responses. First, we used a single, one-hour pulse of the temperature stress (12 °C or 32 °C, compared to 22 °C controls) instead of the longer exposures used in most studies e.g. [10, 11, 27–29]. Long exposures to a stressful temperature significantly affect many plant processes (e.g. growth), leading to complications in determining both the true rates of genetic change [10] and, if it were to be explored, the proximal cause of any change in rate. Second, we timed the one hour window of exposure so as to expose the flowers (that we would later pollinate) while male and female gametophyte development (or the end stage of meiosis) was occurring. This is a highly sensitive period [24, 25], and in one of the few studies to test the effects of short exposures to temperature stress, even one hour (at 4–5 °C) was found to have significant effects on male meiosis, and longer exposures caused reproductive sterility [24, 30]. Third, we have delayed the timing of the exposure to a later stage of the vegetative life-cycle (36 or 41 days after sowing, (DAS)), since flowers from older plants are likely to be more sensitive to a mutation-inducing influence (e.g. [31]). Finally, we did not examine the stressed plants themselves, but only their first-generation progeny, which were the product of a cross between a stressed parent (without a detector transgene) and a non-stressed parent (with the transgene). The impact on rates in the stressed generation is of less interest, at least from a plant breeding perspective; and by using the detector in the non-stressed parent, transgene-related effects are less likely. In the progeny of younger plants there was either no effect of heat and cold (on MR) or, in the case of SMs, the effects were rare and stochastic. For the progeny of older plants, while variation in rates of MR and SMs was also infrequent, when there was variation, it followed a consistent pattern. Rates varied in a “dose-response” manner (being lowest at 12 °C and highest at 32 °C) and this was most obvious (or in the case of MR, only apparent) when the stressed parent was female. While any conclusions must be tempered by the fact that responses were infrequent, the results suggest that the level of stochasticity in inherited genetic responses to heat or cold stress can be context-dependent, possibly reflecting life-cycle stages in the parental generation that are variably stress sensitive.

## Methods

### *Arabidopsis* transgenic lines

Meiotic recombination detector lines 3154, 3158 and 3162 were a gift from Avraham A. Levy (Department of Plant Sciences, Weizmann Institute of Science, Israel). In line 3154 and 3158, the GFP and RFP markers are on chromosome 3 with a genetic distance of 16 and 18 cM respectively between the markers. In line 3162, the GFP and RFP markers are on chromosome 5 and are separated by 21 cM with a centromeric region in between. MR rates were scored based on the segregation of green and red fluorescent markers, which are expressed under a seed specific napin promoter [32].

Base substitution detector lines 166_G→T_ and 1390_T→C_, were a gift from Igor Kovalchuk (University of Lethbridge, Canada) and Anna Depicker (Ghent University, Belgium), respectively [33, 34]. Transgenic intra-chromosomal recombination (ICR) lines (651), carrying an inverted repeat of the *uidA* gene was a gift from Barbara Hohn (Friedrich Miescher Institute, Switzerland) [35]. Transgenic ICR line (R2L1), carrying inverted catalase introns in *uidA* gene, and frameshift detector line (G10), were a gift from Francois Belzile (University of Laval, Canada) [36, 37]. The transposition detection line *Tag1* was a gift from Nigel M. Crawford (UC San Diego, USA) [38].

### Plant growth conditions

Seeds were surface sterilized with 70% ethanol, followed by 0.5% bleach treatment for 3 min. Subsequently, the seeds were washed thrice with sterile water and plated on autoclaved Murashige and Skoog media (MS, with 3% sucrose), pH 5.7, containing 0.05% Plant Preservative Mixture (Biogenuix Medsystem Pvt. Ltd., New Delhi, India). The seeds were incubated at 4 °C in the dark, for synchronized germination. After 48 h, the plates were shifted to a growth chamber (Percival CU-36 L6), with a uniform light intensity of 8000 lx (16-h light/8-h dark cycle), a temperature of 22 °C and 80% humidity. Three-week old seedlings were transferred from MS plates to soil and continued their growth inside the chamber in the same conditions. The soil mixture consisted of equal proportions of garden soil, peat, perlite, and vermiculite (Keltech Energies Ltd., Bangalore, India).

### Experimental procedure for crossing and collecting seeds

**Fig. 1:**
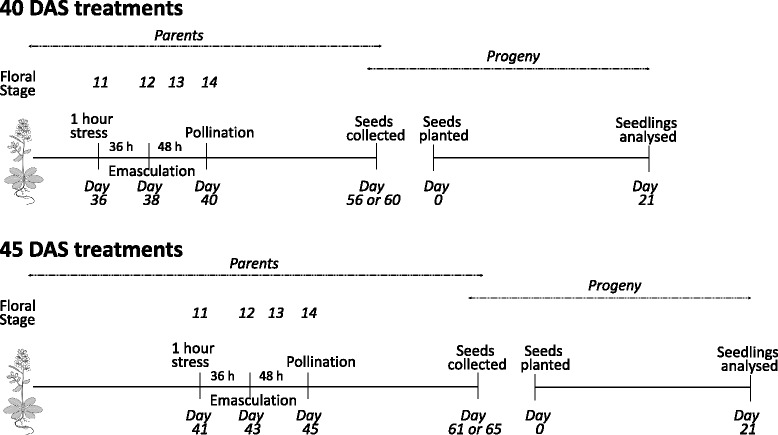
The timing of procedures leading up to the measurement of rates of meiotic recombination (MR, in seeds) or somatic mutations (SMs, in seedlings) in this study. One of the parents of the analysed seeds/seedlings was stressed by shifting wild type plants (Columbia/C24) grown at 22 °C to either 12 °C or 32 °C for one hour at either 36 (or 41) days after sowing (DAS). 36 h later, at 38 (or 43) DAS, we selected some of the flowers that were at Floral Stage 12, and emasculated them. This meant that these flowers were at Stage 11 when the stress was applied (and thus undergoing male and female gametophyte development). 48 h after emasculation, at 40 (or 45) DAS, when the emasculated flowers were now at Stage 14 we (i) pollinated the emasculated flowers with pollen collected from non-emasculated Stage 14 flowers that were on unstressed detector line plants that had been raised, for 40 (or 45) DAS at 22 °C; or (ii) collected pollen from the non-emasculated flowers of Stage 14, stressed, wild type flowers, and used this to pollinate Stage 14 flowers that were on unstressed, detector line plants that had been raised, for 40 (or 45) DAS at 22 °C. In all cases, seeds were collected 16 (for SMs) or 20 (for MR) days after pollination, and either used for analysing rates of MR directly, or planted and grown for a further 21 d, after which the seedlings were analysed for rates of SMs. Coloured threads were used to identify plants of different ages, plants grown at different temperatures, and to differentiate between emasculated, non-emasculated and crossed flowers. The timing of stages is based on data presented in the literature [52, 55, 59]

The procedures for preparing plants prior to analysis are described in Fig. 1. Between 10 and 25 crosses were performed in three independent replicates. For MR rate scoring, the seeds obtained from the crosses were carefully examined under a stereo-zoom fluorescence microscope using GFP and RFP specific filters (Additional file 1: Figure S1). To score somatic mutation rates, a GUS assay was performed on three-week old seedling to score reversion rates (below).

### Histochemical staining for scoring GUS reversions

We followed the protocol of Bashir et al. 2014 [39] and Singh et al. 2015 [20] for histochemical staining of intact three-week old seedlings. Blue spots (Additional file 2: Figure S2) reflect wild type GUS reversions of FSMs, ICR events, base substitutions, and transposition events. Specifically: for FSMs, the detector line carries mononucleotide repeats of G10 within the *uidA* gene and either a single deletion or insertion of two nucleotides would restore the reading frame thereby enabling GUS activity [37]. For ICRs (for which two lines were used) Line 651 (accession C24) carries a 566 bp overlapping GUS sequence in an inverted orientation [35]. Line R2L1 (accession Columbia) carries an inverted catalase intron of 418 bp within the *uidA* gene [36]. For both lines, a recombination event within the identical sequences generates a functional *uidA* gene, resulting in GUS activity. For base substitutions, when we scored nonsense mutations, the transgenic detector line 747 was used, and it carries a G-T transversion at 166th position of *uidA* gene [33]; for scoring missense mutations, line M4 with a T-C transition at 1390th position of *uidA* gene was used [34]. For scoring transposition rates, line *Tag1* (background Columbia), which carries a transposable *Tag1* element between CaMV 35S promoter and *uidA,* was used [38]; excision of *Tag1* will result in functional GUS expression. Spots were counted under a light microscope (Leica KL300), and staining was performed in three replicates.

### Statistical analysis

The total number of plants analyzed per population is indicated in the figures. MR rates follow a normal distribution and hence, a Gaussian generalized linear model, with chi-square test, was used. The number of GUS-spots are count data, which is the reason a Poisson generalized linear model (GLM) with the log link function was chosen [40]. The linear predictors were the different temperatures (12 °C, 22 °C and 32 °C) and, in the case of comparisons of controls, whether the wild type parent was female or male.

In all GLMs, the data from groups were used for several comparisons. Correction for multiple testing was done to maintain the family-wise error rate at 5% [41]. Therefore, we adjusted *P* values with a single-step method that considers the joint multivariate *t* distribution of the individual test statistic [42]. The results were reported with the two-sided *P* value adjusted for multiple comparisons. All statistical analyses were carried out in R [43]. To adjust the *P* values for multiple testing, the R package *multcomp* was used with the test specification “single-step” [42]. Graphs were produced using the ggplot2 package [44] and edited for visual clarity using Pixelmator and Powerpoint.

## Results and discussion

### Impact of temperature stress on rates of meiotic recombination

**Fig. 2:**
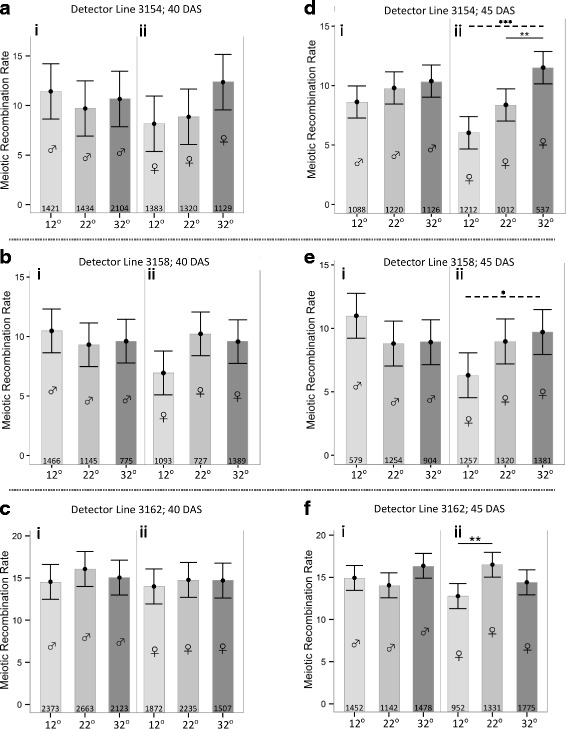
MR rates in the progeny (seeds) from reciprocal crosses of heat- (one hour at 32 °C) or cold- (one hour 12 °C) stressed wild type parents (male or female, as indicated by gender symbols) with one of three detector lines: 3154 (*top row*; **a**, **d**); 3158 (*middle row*; **b**, **e**) and 3162 (*bottom row*; **c**, **f**). 22 °C controls for each treatment are also included. Crosses were carried out on wild type parents who were, at the time of pollination, aged 40 days after sowing (DAS; LHS graphs) or 45 DAS (RHS graphs). Numbers at the bottom of each column indicate the number of seeds analyzed. Point predictions and 95% confidence intervals for MR rates are drawn. Solid lines represent any significantly different comparisons *within* a specific treatment of the 12 °C or 32 °C rates to the 22 °C control (e.g. *within* **d**ii); dashed lines represent any significantly different comparisons, within a specific treatment, of 12 °C and 32 °C rates (e.g. *within* **d**ii or **e**ii). The only other type of comparison made was between the rates of the male and female control treatments for a given detector line and parental age (no differences were found). * – *P* < 0.05; ** – *P* < 0.01; *** – *P* < 0.001; no asterisked line – no significant difference. *P* values are corrected for multiple testing

To examine if a one hour temperature stress (12 °C or 32 °C), applied separately to developing male and female gametes of *Arabidopsis*, would have an impact on MR rates in the first-generation progeny, we crossed stressed wild type parents with one of three unstressed *Arabidopsis* lines (3154, 3158, 3162) homozygous for GFP and RFP markers expressed under a seed specific promoter. This seed based assay to score MR rates relies on the segregation of different fluorescent markers in the subsequent generation [32]. The responses, based on the analysis of 51,278 seeds, differed markedly depending on whether the parents were stressed at 36 or 41 days after sowing (referred to as “40 DAS” and “45 DAS”, the times at which they were pollinated, four days post-stress), and, for 45 DAS progeny, on whether the stressed parent was male or female. In the progeny of both male and female 40 DAS plants, temperature stress had no impact on MR rates (Fig. 2a-c) in the three lines tested, when compared to the progeny of plants constantly maintained at 22 °C. Likewise, temperature stress had no impact for the progeny of 45 DAS plants, when the stressed parent was male (Fig. 2di, ei, fi). When the stressed parent was female however, the 45 DAS rates in all three lines, increased with temperature (Fig. 2dii, eii, fii). This was evidenced by a significant increase in rates between 12 °C and 32 °C (Fig. 2dii, eii), and/or a rise between 12 °C and 22 °C (Fig. 2fii), or 22 °C and 32 °C (Fig. 2dii).

Previous studies of the effects of heat and cold on MR rates in plants have noted rates that increased [14–17], decreased [15, 18], or remained unchanged [19]. The only one of these studies to examine *Arabidopsis* [17] reported that recombination in pollen tetrads correlated positively with temperature, as we saw for the progeny of plants whose female parents were exposed later (the progeny of 45 DAS plants). That study, unlike the other studies, also restricted the duration of exposure to the extreme temperatures, since plants were exposed only after they had begun to bolt [17]. This could limit the opportunity for plants to adapt to the stressful stimulus. We took this idea further by restricting the duration to a single one-hour exposure, and then pollinating emasculated flowers that would have been, at the time of exposure, undergoing gametophyte maturation, or the end stage of meiosis (periods when stress sensitivity is at its highest [24, 25, 45]). This might explain why a one-hour exposure was sufficient to induce a response in our plants. Other temperatures responses are associated with longer exposures: Vernalisation in *Arabidopsis* is, for example, experimentally induced by weeks of exposure to cold (e.g. [46]).

The specificities of the observed responses (i.e. being seen only in the progeny of the older plants, and where it was the female parent that was exposed) have some commonality with previous findings. For example, in one study, MR rates in *Arabidopsis* increased with the developmental age of the shoots upon which the pollinated flowers were located, and, ageing was found to affect (increase) MR rates during female, but not male, gametophyte development in *Arabidopsis* [31]. Several studies have shown that, as in most species, male MR rates in *Arabidopsis* and other plants are higher than female rates (by up to nearly a factor of two; [47–49]), possibly due to the longer duration of prophase in male meiosis [48]. When we include responses other than those involving MR, other studies complement the picture. Transgenerational responses to stress and ageing have been shown, by various indicators, to have a stronger contribution from the female parent (e.g. [20, 50]). This has been argued as being due to the female’s greater contribution to the cytoplasm of the zygote, thus making it the primary carrier of the epigenetic factors that are thought to play a major role in heritable stress responses, including those that show a uniparental bias [9, 50, 51]. In our case, given that male and female meiosis in *Arabidopsis* are slightly out of phase [52], the sex-biased difference in responsiveness could also be due to the one-hour exposure catching the female gametophytes at a more vulnerable stage.

### Somatic mutations

To determine the rates of four types of SMs in the progeny of heat- or cold-stressed plants, we employed a similar strategy to that used for measuring MR rates, except that the various genetic events were detected by the counting of blue dots, each reflecting a reversion of the GUS gene by the mutation [33] in three-week old seedlings. As with the MR experiments, hot or cold stress was applied to either the male and female wild-type parent, and the reciprocal, unstressed parent carried the GUS reporter. As will be described in detail below, while the overall pattern of responsiveness might be described as “rare and “stochastic” [7], a consistent pattern, with several similarities to that seen with MR responses, did emerge in seedlings from the 45 DAS progeny.

### Rates in the progeny of control plants showed a strong parent-of-origin bias

**Fig. 3:**
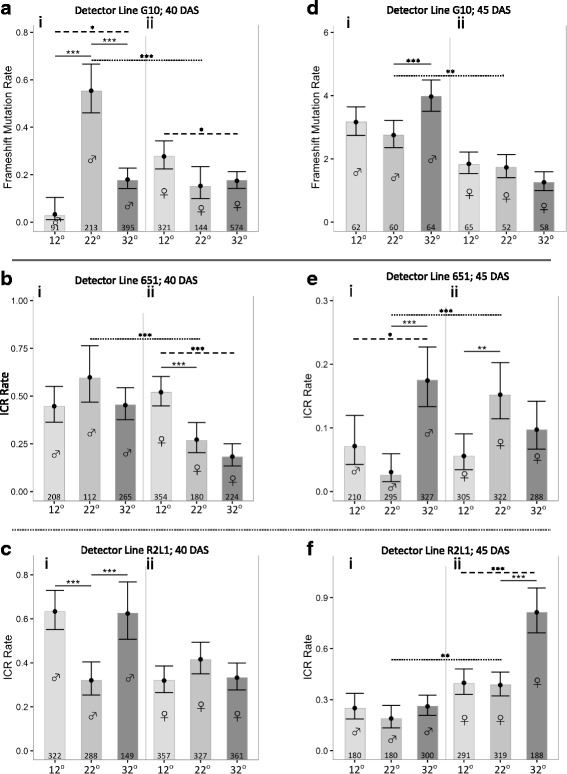
The rates of two types of somatic mutations (FSMs; *top row*; **a**, and **d**) and ICRs (middle and bottom rows; **b**, **e**, **c**, **f**) in the progeny (seedlings) from reciprocal crosses of heat- (one hour at 32 °C) or cold- (one hour 12 °C) stressed wild type parents (male or female, as indicated by gender symbols) with one of three detector lines: G10 (FSMs); or 651 and R2L1 (ICRs). 22 °C controls for each treatment are also included. Crosses were carried out on wild type parents who were, at the time of pollination, aged 40 DAS (LHS graphs) or 45 DAS (RHS graphs). Numbers at the bottom of each column indicate the number of seedlings analyzed. Point predictions and 95% confidence intervals for SM rates are drawn. Solid lines represent any significantly different comparisons *within* a specific treatment of the 12 °C or 32 °C rates to the 22 °C control (e.g. in 3 Ci); dashed lines represent any significantly different comparisons, within a specific treatment, of 12 °C and 32 °C rates (e.g. *within* **a**ii). The only other type of comparison made was between the rates of the male and female control treatments for a given detector line and parental age. For these, any statistically significant difference is indicated by a dotted line (e.g. *between* **f**i and **f**ii). * – *P* < 0.05; ** – *P* < 0.01; *** – *P* < 0.001; no asterisked line – no significant difference. *P* values are corrected for multiple testing

**Fig. 4:**
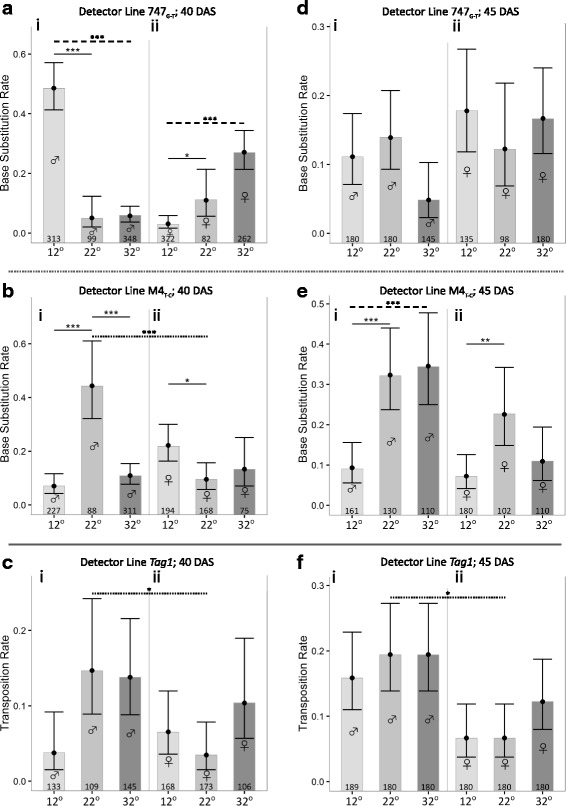
The rates of two types of somatic mutations (base substitutions; top and *middle rows*; **a**, **d**, **b**, **e**) and transpositions (*bottom row*; **c**, **f**) in the progeny (seedlings) from reciprocal crosses of heat- (one hour at 32 °C) or cold- (one hour 12 °C) stressed wild type parents (male or female, as indicated by gender symbols) with one of three detector lines: 747_G–T_ or M4_T-C_ (base substitutions) or *Tag1* (transpositions). 22 °C controls for each treatment are also included. Crosses were carried out on wild type parents who were, at the time of pollination, aged 40 DAS (LHS graphs) or 45 DAS (RHS graphs). Numbers at the bottom of each column indicate the number of seedlings analyzed. Point predictions and 95% confidence intervals for SM rates are drawn. Solid lines represent any significantly different comparisons *within* a specific treatment of the 12 °C or 32 °C rates to the 22 °C control (e.g. *in* **b**i); dashed lines represent any significantly different comparisons, within a specific treatment, of 12 °C and 32 °C rates (e.g. *within* **a**ii). The only other type of comparison made was between the rates of the male and female control treatments for a given detector line and parental age. For these, any statistically significant difference is indicated by a dotted line (e.g. *between* **f**i and **f**ii). * – *P* < 0.05; ** – *P* < 0.01; *** – *P* < 0.001; no asterisked line – no significant difference. *P* values are corrected for multiple testing

Before beginning the main analysis, one interesting feature of the SM rates in the progeny of control plants needs to be discussed. While there were no significant differences between any of the reciprocal cross controls (i.e. male wild type x female detector-line, and vice versa, at 22 °C) in the MR experiments, this was not the case with the SM controls (Figs. 3a–4f). In eight of the twelve possible comparisons, there was a significant difference (shown by dotted lines in Figs. 3a, b, d–f and Fig. 4b, c, f) and each mutation type exhibited at least one significant difference. In six of the eight significant differences, SM rates were higher when the cross involved a male wild type line x a female detector line. With respect to the male Columbia accession line used, it might have a naturally high rate of mutation, either intrinsically or by virtue of a higher copy number [53]. As to whether the female line is responsible, if so, this could not be due to a higher copy number because all the detector lines only have a single copy of the genome (references in Methods section: ‘*Arabidopsis* transgenic lines’). Also, any contribution from the transgene in the female line is argued against by a previous study where the mutation rate was found to decrease as transgene expression rose [33]. One possibility, however, for the female line, is via its greater contribution, relative to the paternal line [51, 54], of cytoplasm to the zygote (and thus, potentially, more epigenetic factors).

Comparisons of the rates in progeny of 40 and 45 DAS control plants also demonstrated that the age of the parent plants can be an important influence for some types of mutations (principally for FSMs and transpositions, as we reported previously [20]). While we have not analysed this statistically here, it is clear, that, as in the previous study, FSM rates increased dramatically with age (Fig. 3a, d); however, unlike before, transposition rates did not respond to age (Fig. 4c, f). This could be due to the fact that though the same detector line was used in both studies, the age difference between the youngest and oldest parents in this study was less than half of that used previously. It should also be noted here that, in this study, while the parental plants differed in age, the stressed flowers that were one source of the male or female gametes, were always stressed at approximately the same stage (Floral Stage 11). This is possible because the individual flowers of the inflorescences of *Arabidopsis* have a highly staggered pattern of maturation [31, 55].

### Responses to heat and cold in progeny of 40 DAS parents were rare and mostly stochastic

**Fig. 5:**
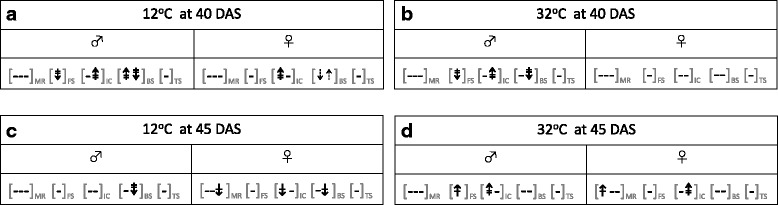
These figures present, in a more consolidated way, one subset of the significant differences shown in detail in Figs 2, 3 and 4 (i.e. those shown using solid lines in those graphs, thus showing significant differences, within any one treatment, between either the 12 °C and 22 °C rates, at 40 and 45 DAS (**a** and **c**, respectively) or the 32 °C and 12 °C rates at 40 and 45 DAS (**b** and **d**, respectively). As in the previous figures, the gender symbols indicate the gender of the wild type parent that experienced the stress (the other parent was an unstressed detector line). The subscripted abbreviations MR, FS, IC, BS, and TS refer to, respectively: meiotic recombination, frameshift mutations, intrachromosomal recombination, base substitutions and transpositions. These genetic events were scored with either one, two or three detector line/s, and where more than one detector line was used, the sequence of the symbols within any one set of brackets reflects the sequence of the detector lines shown in Figs 2, 3 or 4 (i.e for the MR set shown in Fig. 5, the three symbols refer to, in order: lines 3154, 3158 and 3162; the ICR lines in sequence are: 651; R2L1; and the BS lines are 747 and M4). The up and down arrows indicate, respectively, that (relative to the control rate) in the progeny of a stressed parent, the rate of the designated genetic event was higher or lower. The dotted, single- and double-barred arrows indicate that the significant differences had, respectively, the following levels of significance: *P* < 0.05; *P* < 0.01; and *P* < 0.001. A dashed line indicates that there was no significant difference

Our main interest was how heat (32 °C) or cold (12 °C) affected the rates of the various mutations relative to the control (22 °C). The significant differences for progeny from the 40 DAS parents are (a) shown in the individual graphs (Figs. 3a–c, 4a–c, solid line comparisons), and, (b) summarised in Fig. 5a and b. Only 9 out of 36 possible responses were significant, and there was little pattern amongst these nine, either with respect to the temperature the parents were exposed to, or to the sex of the parent that was stressed (Fig. 5a and b). Only in two limited cases, was there consistency: when the stressed parent was male, rates of FSMs decreased with both cold and heat (Fig. 5a and b), while rates of ICRs always increased with cold and heat in one of the two detector lines used (R2L1; Fig. 5a and b).

Collectively, the responses of the progeny of 40 DAS parents can be characterised as “rare and stochastic”, a phrase used previously to describe how IRC rates varied in the progeny of *Arabidopsis* plants subjected to a wide range of stresses, including heat and cold [7]. In that study, and others (e.g. [56], the authors argue that such stochasticity usefully augments an organism’s flexibility in responding to environmental stresses. Some of the variation seen here and in the study overall, could arise also from the different detector lines used. For example, for base substitutions, one line, 747, was used to score T-G transversions, while line M4 was used to score C-T transitions. Given that methylated cytosine is more prone to deamination in the genome [57], and levels of methylation increase throughout plant development [58], there could be an increase in C-T transition rates between 40 and 45 DAS, thus resulting in a proportionately higher change in the rate of transitions in the progeny.

### Responses to heat and cold in progeny of 45 DAS parents were rare but not stochastic

In the progeny of the 45 DAS parents, responses to both heat and cold were rare, but there was consistency amongst the responses that were significant. The significant differences for progeny from the 45 DAS parents are (a) shown in the individual graphs (Figs 3d–f, 4d–f, solid line comparisons), and, (b) summarised in Fig. 5c and d. Only 8 out of 36 possible responses were significant, but whenever a response was seen to cold or heat, the rates always decreased or increased, respectively (Fig. 5c, and d). Within the subset of significant responses, there was a slight parent-of-origin bias: 5 of the 8 responses occurred when the stressed wild type parent was female (Fig. 5c and d).

While the idea that we are seeing an authentic (non-coincidental) pattern of responses in the progeny of the older plants must be tempered by the overall infrequency of responses, several other observations boost the argument that it is. First, the pattern is also supported when we compare the 12 °C and 32 °C rates: in all three of the cases where there is a significant difference, the rate is higher at 32 °C (comparisons shown by dashed lines in Figs. 3e, f, and Fig. 4e). Second, when all the significant differences for the progeny of 45 DAS parents are considered together, they hint at the same type of ‘dose-response’ pattern seen with MR rates in the progeny of 45 DAS (female-stressed) parents: i.e. rates, if they respond at all, increase with temperature, and are more likely to do so when the stressed parent is female.

## Conclusions

From a reading of the collective research on how rates of MRs and SMs respond in the progeny of stressed parent plants, it would easy to agree with the conclusion that responses are, overall, “stochastic” [7]. And, likewise, that it is the capacity of stress to enable random genetic variation that is important, rather than any responses being adaptively appropriate in their own right. The findings of this study however point to another possibility. In certain circumstances, for example those prevailing in our stressing of older plants, the rates of both MRs and SMs might respond in a more consistent manner, increasing with temperature. Our protocol introduced several novel approaches: applying the stress for just one hour, at a stage (1) when transmittable genetic and epigenetic material is most vulnerable to stress (i.e. during gamete and gametophyte maturation; [24, 25, 45]) and (2) when, in the case of the older parental plants, genetic and epigenetic repair mechanisms are likely to be less effective [21, 23, 25, 31]. While we cannot rule out, as yet, that the consistent patterns we have seen might themselves be stochastic coincidences, they are intriguing enough, and have sufficiently significant implications for both plant breeding and general biology, to warrant critical consideration and further study.

## Additional files

Additional file 1: Figure S1. Crossing scheme depicting the generation of heterozygous MR tester lines. (A). Cross between MR detector lines and wild type plants exposed to different temperatures. (B). Seeds under RFP specific filter and (C). Under GFP specific filter. (D). Merged image of both showing four different populations, where green only and red only represent recombinant seeds. (PDF 150 kb)

Additional file 2: Figure S2. GUS reversion event resulting in a blue spot in the leaf of a three-week old seedling. (PDF 88 kb)

## Abbreviations

cM: Centimorgan
DAS: Days after sowing
DSBs: Double strand DNA breaks
FSM: Frameshift mutation
GFP: Green fluorescent protein
GLM: Generalized linear model
*GUS*: β-Glucuronidase gene
ICR: Intrachromosomal recombination
MR: Meiotic recombination
MS: Murashige and Skoog media
RFP: Red fluorescent protein
