# Supplementary figures and images for: Brief temperature stress during reproductive stages alters meiotic recombination and somatic mutation rates in the progeny of Arabidopsis
Source: BMC Plant Biol. 2017 Jun 14;17:103. doi: 10.1186/s12870-017-1051-1 (PMC5471674; doi:10.1186/s12870-017-1051-1)

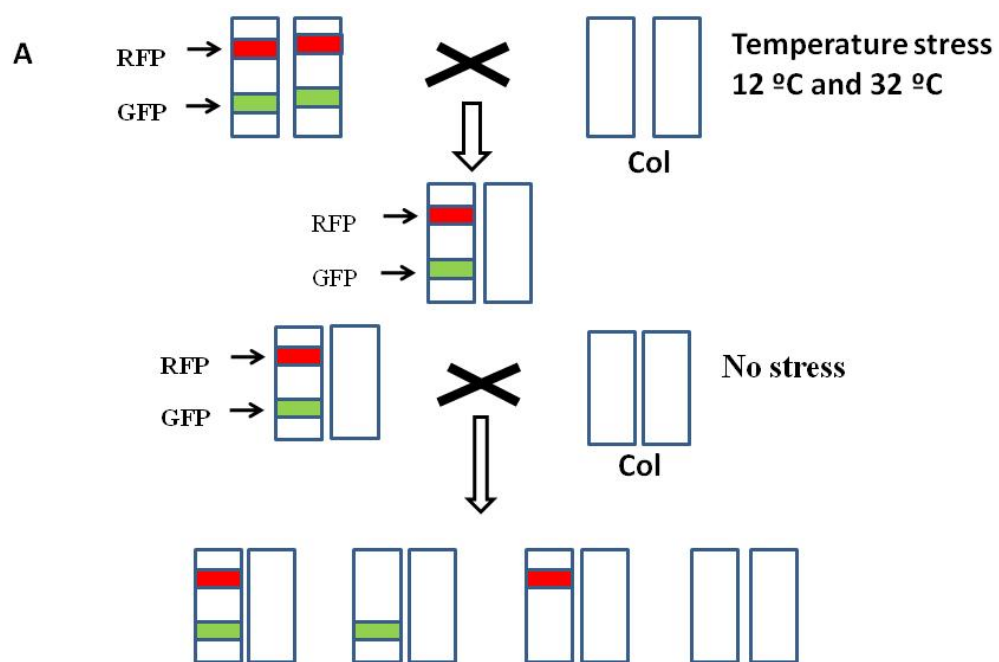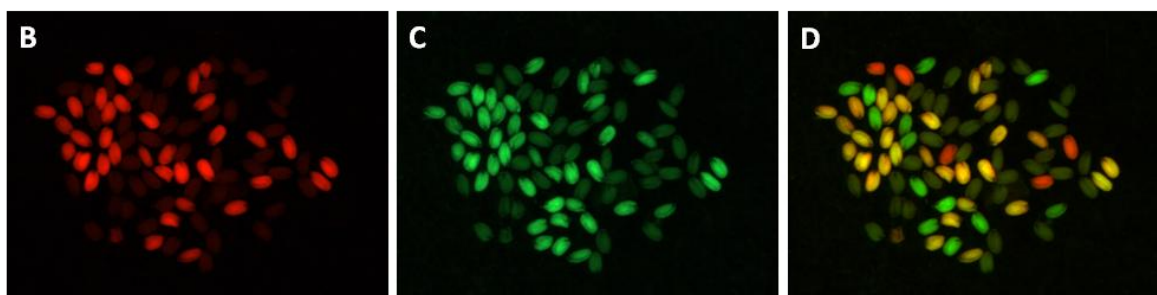

Supplement: Supplementary file 1 — Crossing scheme depicting the generation of heterozygous MR tester lines. (A). Cross between MR detector lines and wild type plants exposed to different temperatures. (B). Seeds under RFP specific filter and (C). Under GFP specific filter. (D). Merged image of both showing four different populations, where green only and red only represent recombinant seeds. (PDF 150 kb) [file 12870_2017_1051_MOESM1_ESM.pdf]

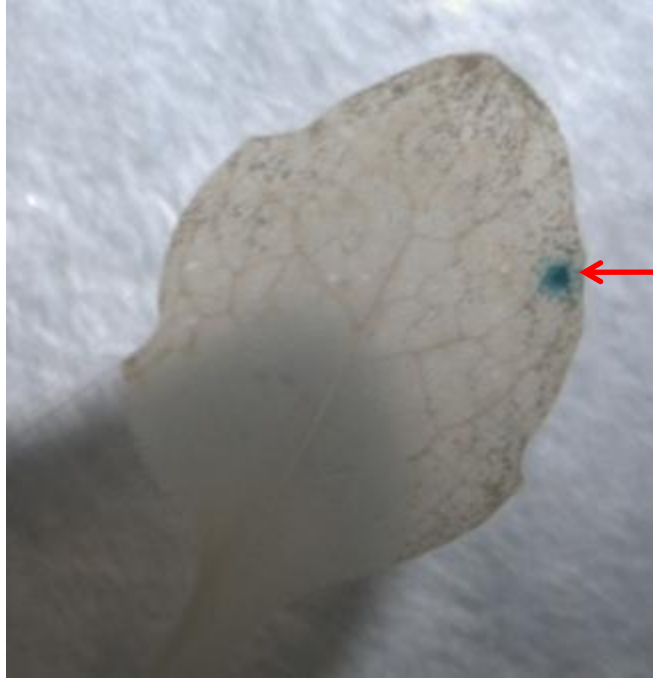

Supplement: Supplementary file 2 — GUS reversion event resulting in a blue spot in the leaf of a three-week old seedling. (PDF 88 kb) [file 12870_2017_1051_MOESM2_ESM.pdf]
